# The Great Ordeal

**Published:** 1893-08

**Authors:** W. S. Elliott

**Affiliations:** Sag Harbor, N. Y.


ARTICLE V.

### THE GREAT ORDEAL.

BY DR. W. S. ELLIOTT, SAG HARBOR, N. Y.

Patient: Doctor, I feel terribly nervous about having my teeth taken out; I have been trying to screw up my courage for more than a year, and now I am suffering so much pain that I suppose I must submit to the dreadful ordeal. Will you please look at them, and tell me if it will be a painful operation—and ought I to take gas?

Doctor: Please take the chair; I will look them over, and advise you to the best of my judgment. * * * Well! I am surprised that you should desire to lose any of your teeth. It is true, several are decayed; one has an exposed and inflamed pulp; another is broken off nearly to the gum line, and the gums and mouth generally are in a diseased condition; but these are not sufficient reasons for extraction. Do you not know that it is within the province of the dentist, who has given close study to these subjects, to treat all these affections, and thus restore every tooth and the mouth to healthy conditions.

Patient: But, doctor, they pain me, and I cannot bear the agony another day.

Doctor: You need not; allow me to serve you from the standpoint of professional ability, and I trust you will thank me for the results. This little pellet I will seal in the cavity of exposure, preliminary to further treatment. Doubtless your past dental experience has been disappointing, and you are impressed with the idea that nothing can be done but to extract. Surely it would be deep humiliation if, after so many years of carful study and practice, I was obliged to acknowledge that no progress has been made towards saving the teeth. Old time methods and the lack of knowledge of the true nature of disease, indeed, did often fail, and even now there are many who are plodding along in the old weedy paths of ignorance and indifference. But new light has fallen on the profession in these latter days, and greater possibilities are realized. The microscope has opened up new revelations; chemistry has unfolded to the understanding much that has heretofore been hidden relative to the composition of matter; electricity shows its play of activities in connection with living tissues; new remedies have been discovered, and new methods of manipulation adopted. Bacteriology and the science of therapeutics are revolutionizing the practice of dental medicine. Scarcely a month or a week goes by but some new discovery is announced, and what was once only hoped for is now happily and surely accomplished.

Now, while I have been talking, your countenance assures me that the pain has subsided and you are comparatively comfortable. The medicine has had a soothing influence. and it will bridge you over till my time and your opportunity will permit us to follow out the line of treatment indicated.

Patient: But the broken tooth—what will you do with that? I suppose in this instance you will resort to extraction?

Doctor: Not at all. I will build on the roots a crown of gold which will be to you a joy forever; and across these vacant spaces I will span a bridge of gold and porcelain that will enable you to masticate more thoroughly, and so relieve the natural teeth from the undue work and strain that they are now subjected to. Your gums and the mouth generally will receive proper medicinal treatment, whereby ease will be instituted instead of disease and discomfort. When all this is accomplished I will demand of you your cooperation in the maintenance of a condition of permanent health. I will insist that you be diligent in brushing the teeth and cleansing the mouth night and morning; the microbes will thus be vanquished, and victory be written on the shield of your endeavor.

Patient: Doctor, I thank you for your words of encouragement. This is all new to me. Had I been aware of the possibilities you speak of I would not have suffered these many months with worriment and pain. I have heard incidentally of the vast strides made in dental science, but I have not before realized the fact as you have set it before me, and I now see that this advanced position is only attained by those who love their profession, and who are foremost in their endeavors to secure the highest good attainable.

Doctor: Yes; colleges have been instituted for systematic instruction; societies are formed for the promulgation of knowledge and the cultivation of social intercourse among the members. Thus, fraternity of feeling is fostered, jealousies annihilated, and interchange of views and experiences made, to the betterment of all who participate. He who is not progressive is retrogressive, since such an one will be left far behind in the race for the championship.

Patient: Encouraged as I am, I will be guided by your judgment and advice. There is one thing, however,

I would like to speak of: What will be the probable cost of all this? I am not like Cleon, who "hath an hundred acres;" therefore I would not deem it an improper question to ask,

Doctor: It is impossible to definitely estimate it, but whatever it may be it will be money well invested, and it will pay you a better interest than do many of the indulgencies of your every-day life. As you trust me in my ability to serve you professionally, so you will confide in me to do you justice in the fee. Now, in the confidence of professional intercourse, we will enter on our duties, and will name a day and an hour which will be devoted to you exclusively, and let us trust that no trivial circumstance will intervene to prevent a prompt fulfilment of the appointment.—*Items of Interest.*

